# Pyrosequencing of the *Camptotheca acuminata *transcriptome reveals putative genes involved in camptothecin biosynthesis and transport

**DOI:** 10.1186/1471-2164-12-533

**Published:** 2011-10-30

**Authors:** Yongzhen Sun, Hongmei Luo, Ying Li, Chao Sun, Jingyuan Song, Yunyun Niu, Yingjie Zhu, Liang Dong, Aiping Lv, Enzo Tramontano, Shilin Chen

**Affiliations:** 1 The Key Laboratory of Bioactive Substances and Resources Utilization of Chinese Herbal Medicine, Ministry of Education, Institute of Medicinal Plant Development, Chinese Academy of Medical Sciences & Peking Union Medical College, Beijing 100193, P. R. China; 2 Institute of Basic Theory, China Academy of Traditional Chinese Medicine, Beijing, China; 3 Department of Applied Sciences in Biosystems, University of Cagliari, Monserrato (Cagliari), Italy

## Abstract

**Background:**

*Camptotheca acuminata *is a Nyssaceae plant, often called the "happy tree", which is indigenous in Southern China. *C. acuminata *produces the terpenoid indole alkaloid, camptothecin (CPT), which exhibits clinical effects in various cancer treatments. Despite its importance, little is known about the transcriptome of *C. acuminata *and the mechanism of CPT biosynthesis, as only few nucleotide sequences are included in the GenBank database.

**Results:**

From a constructed cDNA library of young *C. acuminata *leaves, a total of 30,358 unigenes, with an average length of 403 bp, were obtained after assembly of 74,858 high quality reads using GS *De Novo *assembler software. Through functional annotation, a total of 21,213 unigenes were annotated at least once against the NCBI nucleotide (Nt), non-redundant protein (Nr), Uniprot/SwissProt, Kyoto Encyclopedia of Genes and Genomes (KEGG), and *Arabidopsis thaliana *proteome (TAIR) databases. Further analysis identified 521 ESTs representing 20 enzyme genes that are involved in the backbone of the CPT biosynthetic pathway in the library. Three putative genes in the upstream pathway, including genes for geraniol-10-hydroxylase (*CaPG10H*), secologanin synthase (*CaPSCS*), and strictosidine synthase (*CaPSTR*) were cloned and analyzed. The expression level of the three genes was also detected using qRT-PCR in *C. acuminata*. With respect to the branch pathway of CPT synthesis, six cytochrome P450s transcripts were selected as candidate transcripts by detection of transcript expression in different tissues using qRT-PCR. In addition, one glucosidase gene was identified that might participate in CPT biosynthesis. For CPT transport, three of 21 transcripts for multidrug resistance protein (MDR) transporters were also screened from the dataset by their annotation result and gene expression analysis.

**Conclusion:**

This study produced a large amount of transcriptome data from *C. acuminata *by 454 pyrosequencing. According to EST annotation, catalytic features prediction, and expression analysis, novel putative transcripts involved in CPT biosynthesis and transport were discovered in *C. acuminata*. This study will facilitate further identification of key enzymes and transporter genes in *C. acuminata*.

## Background

Camptothecin (CPT) was first extracted from the stems of *Camptotheca acuminata *in 1966 and subsequently from *Nothapodytes foetida, Ophiorrhiza pumila*, and *Ophiorrhiza japonica *[1]. CPT exhibits clinical anti-tumor activity by inhibiting DNA topoisomerase I, an enzyme involved in DNA recombination, repair, replication, and transcription [2]. CPT also inhibits the retroviruses, such as the human immunodeficiency virus [3]. Despite its significant clinical use, the main source of CPT is still from its extraction from *C. acuminata*. However, the quantity is quite limited and cannot meet worldwide demand. Studies on the molecular mechanism of CPT biosynthesis have long been hindered by the lack of transcriptome and genome information for *C. acuminata *and other CPT-producing plants. Therefore, it is necessary to obtain transcriptome data and screen candidate transcripts involved in CPT biosynthesis to further understand the CPT biosynthetic pathway.

**Figure 1:**
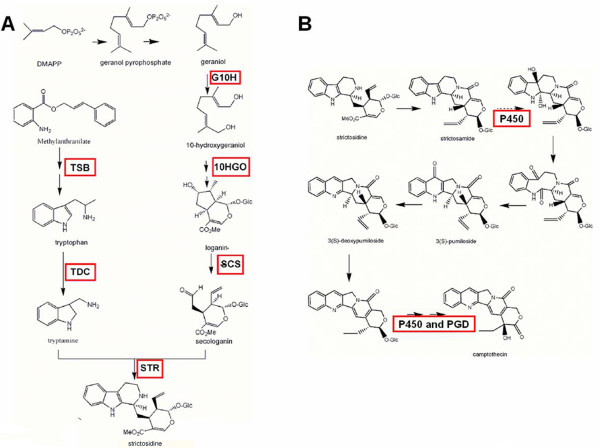
**Biosynthetic pathway of CPT from DMAPP to strictosidine and from strictosidine to CPT in *C. acuminata***. (A) The upstream pathway for the synthesis of backbone strictosidine. (B) The proposed branch pathway of CPT biosynthesis (steps after strictosidine synthesis). TSB: β-subunit of tryptophan synthase; TDC: tryptophan decarboxylase; G10H: geraniol-10-hydroxylase; SCS: secologanin synthase; STR: strictosidine synthase; 10-HGO: 10-hydroxy geraniol oxidoreductase. PGD: putative strictosidine β-D-glucosidase. The arrow with the dotted shaft represents the step that was presumed in the study to be catalyzed by a CYP450.

CPT is synthesized through a modified terpenoid indole alkaloid (TIA) pathway. The upstream biosynthesis pathways for all the TIA products are similar among alkaloid-producing plants, and involve a strictosidine backbone (Figure 1A). Over recent decades, several enzymes in the process of strictosidine biosynthesis in *C. acuminata *have been isolated and functionally identified. Among them are tryptophan synthase (TSB) [4] and tryptophan decarboxylase (TDC) [5], which are involved in the synthesis of the indole precursor tryptamine, 3-hydroxy-3-methylglutaryl-CoA synthase (HMGR) [6], 1-deoxy-D-xylulose-5-phosphate reductoisomeras (DXR) [7], and 10-hydroxy geraniol oxidoreductase (10HGO) [8] are involved in secologanin synthesis.

G10H and SCS, belonging to the CYP76B6 and CYP72A1subfamilies of cytochrome P450 family respectively, were identified in monoterpenoid biosynthesis from *Catharanthus roseus *[9,10]. The synthesis of strictosidine is finally catalyzed by STR, a committed enzyme for the CPT backbone biosynthesis, which was isolated and identified in *Rauvolfia serpentine*, *C. roseus*, the CPT-producing plant *O. japonica*, and *O. pumila*, in previous studies. However, the genes encoding CaG10H, CaSCS and CaSTR, have not been yet cloned and characterized in *C. acuminata*.

The steps following strictosidine formation (branch pathway) are not very clear and only a proposed biosynthetic pathway based on relative compounds extracted from CPT-producing plants has been reported [11] (Figure 1B). In the proposed pathway, a series of oxidation and hydroxylation reactions are involved in some steps of the pathway which are probably catalyzed by monooxygenases and hydroxylase, belonging to the superfamily of cytochrome P450s [12,13]. Meanwhile, the branch pathway of CPT biosynthesis is unique among TIA pathways because strictosidine is not immediately deglycosylated as in *C. roseus *[14]. However, it requires a glucosidase for glycoside hydrolysis, which likely occurs in one of the last steps of CPT biosynthesis in *C. acuminata *and other CPT-producing plants. At present, the CYP450s and glucosidase involved in CPT biosynthesis have not been studied in *C. acuminata*.

Glandular trichomes in leaves are the main site for CPT accumulation in *C. acuminata *[15]. However, gene expression involved in CPT synthesis was not detected in glandular trichomes but, instead, in epidermal cells and mesophyll cells in *C. acuminata *leaves, which implied the translocation of CPT between organs or cells [16]. Multidrug resistance protein (MDR) transporters, belonging to the ATP-binding cassette (ABC) transporter family, were reported to be responsible for uptake or secretion in alkaloid transportation in some plants [17-19]. Therefore, we hypothesized that MDR transporters are responsible for CPT transportation from other cells to glandular trichomes in *C. acuminata*. At present, no CPT transport mechanism or related genes have been investigated in *C. acuminata*.

Expressed sequence tags (ESTs) analysis has been a primary tool for the discovery of novel genes, based on the traditional Sanger sequencing principle, which is slow and costly for non-model species with little genomic information. The emergence of high throughput platforms, such as pyrosequencing technology [20], enables comprehensive study of the transcriptome for various purposes, such as development study, miRNA identification, and genetic polymorphisms discovery in plants and animals [21-23]. The Roche/454 GS FLX platform, one of the high throughput sequencing platforms, offers the advantages of longer read length and lower cost which is especially suitable for *de novo *transcriptome sequencing aimed at gene discovery and analysis in a specific metabolic pathway [24,25]. Previous studies have indicated that the content of CPT in young leaves is higher than that in old leaves and root [26,27], suggesting that young leaf is an important tissue for the study of CPT biosynthesis and transport. Therefore, cDNA from *C. acuminata *young leaves was subjected to *de novo *transcriptome sequencing to uncover genes involved in CPT biosynthesis and transport, using a Roche/454 GS FLX titanium sequencing platform, a next-generation sequencing system. Based on the sequencing and analysis results, three important genes likely to be involved in the CPT biosynthesis were cloned and analyzed. From data analysis and expression analysis, six cytochrome P450s and one glucosidase gene were found to be candidate genes in the process of CPT biosynthesis. Meanwhile, three MDR transporter genes were also found to be candidate genes involved in CPT transportation.

## Results and Discussion

### 454 sequencing and EST assembly

**Figure 2:**
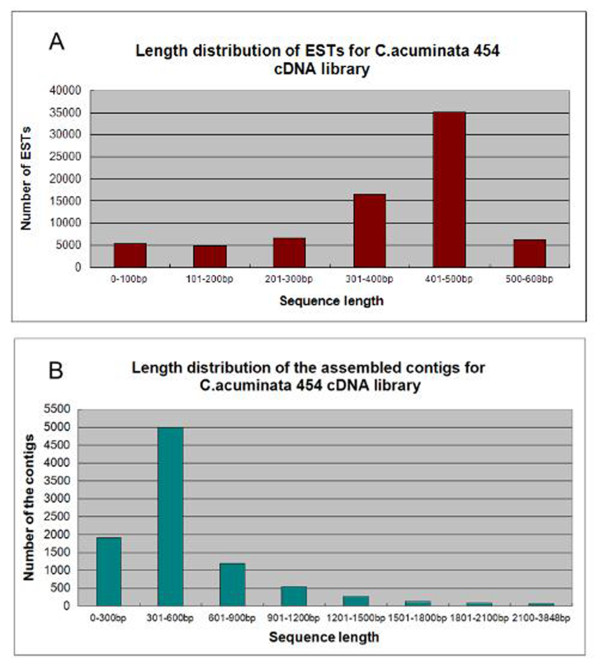
**Primary sequencing results for the cDNA library of *C. acuminata***. (A) Length distribution for ESTs of the 454 dataset. (B) Length distribution of the assembled contigs of the cDNA library.

Through 454 deep pyrosequencing, 74,858 high-quality (HQ, > 99.5% accuracy on single base reads) reads were generated and then submitted to the Sequence Read Archive of NCBI with an accession number SRX033123. The total length of all the reads is 28,746,026 bp, and the average size is 384 bp. After sequence assembly, 30,358 unigenes, with an average length of 403 bp, were generated, including 9,145 contigs and 21,213 singletons. The average coverage was 3.72-fold. The assembled contigs ranged from 96-3848 bp, with a mean length of 525 bp, including 8,485 contigs which were more than 200 bp (about 92.8%). The singletons ranged from 50 bp to 608 bp, with an average length of 351 bp. The length distribution of HQ reads (Figure 2A) and assembled contigs (Figure 2B) are shown for evaluation of the quality of the library. A summary of the sequencing and assembly results is provided in Table 1.

**Table 1:** Summary of *C.acuminata *EST sequencing and assembly

	Numbers of ESTs	Average length (bp)	Total bases (bp)
HQ (high quality) ESTs	74,858	384 ± 125	28,746,026
HQ reads for assembly	51,085	351	17,942,116
Contigs	9,145	525 ± 356	4,799,960
Singletons	21,213	351 ± 137	7,445,387
Unigenes (contigs and singletons)	30,358	403	12,245,347

### Annotation and categorization

A total of 21,213 unigenes (69.87%, 21,213/30,358) were functionally characterized against the NCBI nucleotide (Nt), non-redundant protein (Nr), Uniprot/SwissProt, Kyoto Encyclopedia of Genes and Genomes (KEGG), and *Arabidopsis thaliana *proteome (TAIR) databases [28-32]. An overview of the annotation statistics against public databases (Additional file 1A) and a summary of the most abundant (Additional file 1B) and longest transcripts of the dataset (Additional file 1C) are listed in the supporting information.

To functionally categorize the information in this EST pool, all unigenes were characterized by Gene Ontology (GO) analysis, provided by the TAIR database. A total of 18,172 unigenes were classified into three large categories and forty-five subcategories, based on GO classification [33], accounting for approximately 60% of all the unigenes (Additional file 2).

### Transcripts for proteins involved in the backbone biosynthetic pathway of CPT

#### Putative strictosidine synthesis genes discovered in the dataset

Strictosidine is the precursor and backbone of many TIAs, including CPTs, in plants such as *C. acuminata*. A proposed biosynthetic pathway of strictosidine is shown in Figure 1, and each of the main enzymes present in the dataset is marked with a bold box (Figure 1-A). From the 454 data pool, 521 ESTs representing 20 enzyme genes involved in strictosidine biosynthesis were discovered. Thirteen of these genes had not been previously reported, including the important enzymes geraniol-10-hydroxylase (G10H), secologanin synthase (SCS), and strictosidine synthase (STR) (Table 2). By searching the annotation information from the Nr, Swissprot, and KEGG databases, we found that transcripts of 1-deoxy-D-xylulose-5-phosphate reductoisomerase (DXR), 10HGO, and SCS were presented many ESTs, indicating that they are highly expressed in the young leaves of *C. acuminata*. G10H and TDC were both rare transcripts in the dataset, indicating that they are rarely expressed and are possibly rate-limiting genes in the tissue. The specific annotation information of some putative transcripts against the Nr, Swissprot and KEGG databases is shown in the supporting information (Additional file 3).

**Table 2:** Statistics of putative genes involved in camptothecin biosynthesis^a^

Enzyme code	Name of enzyme	Number of unigenes in cDNA library	Number of ESTs in cDNA library	Number of nucleotides in GenBank
2.3.1.9	acetoacetyl-CoA thiolase	2	15	0
2.3.3.10	HMG-CoA synthase	4	7	1
1.1.1.34	HMG-CoA reductase	2	9	3
2.7.1.36	mevalonate kinase	1	5	0
2.7.4.2	phosphomevalonate kinase	2	2	0
4.1.1.33	mevalonate-5-diphosphate decarboxylase	2	6	0
2.2.1.7	DXP synthase	8	105	0
1.1.1.267	DXP reductoisomerase	1	19	1
2.7.7.60	MEP cytidylyltransferase	0	0	0
2.7.1.148	CDP-ME kinase	3	3	0
4.6.1.12	MECDP synthase	1	1	0
1.17.7.1	4-hydroxy-3-methylbut-2-enyl-diphosphate synthase	1	33	0
1.17.1.2	4-hydroxy-3-methylbut-2-enyl-diphosphate reductase	1	13	0
5.3.3.2	isopentenyl-PP isomerase	2	11	1
2.5.1.10	farnesyl diphosphate synthase	4	13	0
1.14.14.1	geraniol 10-hydroxylase	1	11	0
1.-.-.-	10-HG oxidoreductase	3	84	1
1.3.3.9	secologanin synthase	17	165	0
4.2.1.20	β-subunit of tryptophan synthase	4	4	2
4.1.1.28	tryptophan decarboxylase	1	1	2
4.3.3.2	strictosidine synthase	6	14	0
Total number		66	521	11

Notes: ^a^--Total numbers when annotated against Nr, Swissprot and Kegg databases.

**Figure 3:**
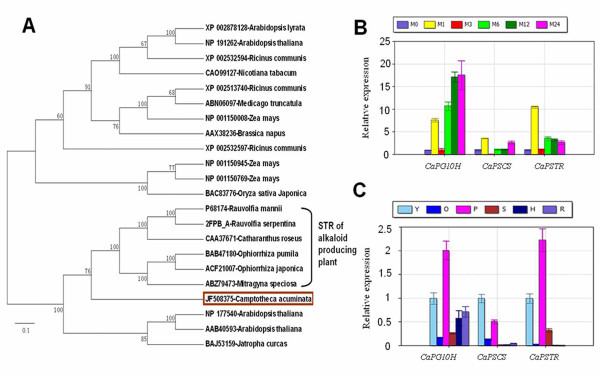
**Phylogenetic tree analysis of strictosidine synthases (STR) and the expression profile detection of *CaG10H, CaSCS*, and *CaSTR***. (A) Protein sequences for 22 STRs were aligned using the ClustalW module and phylogenetic tree was constructed using MEGA 4.0. (B) Relative expression of three genes after induction by MeJA. Expression levels in young leaves without treatment served as controls (M0). M1, M3, M6, M12 and M24 indicate that the treatment times of 1 h, 3 h, 6 h, 12 h and 24 h, respectively. (C) The quantification of three genes involved in CPT biosynthesis in different tissues. Expression levels in young leaves served as controls. Y: young leaves; O: old leaves; P: petioles; S: stems; H: root bark; R: root.

G10H, SCS, and STR are the most important enzymes in the synthesis of strictosidine in TIA-producing plants, including *C. acuminata*. CrG10H, the first CYP450 in CPT synthesis, is a rate-limiting enzyme in the process of TIA synthesis in *C. roseus*. In the 454 dataset, only one read of G10H was found and it had approximately 60% identical to the G10H gene of *C. roseus *and *Swertia mussotii*. Based on the EST sequence, a putative *G10H gene in C. acuminata *(*CaPG10H) *gene was cloned (GenBank ID: JF508378) and analyzed. Similarity analysis of the amino acid sequence showed that *CaPG10H *shared 56% identity to *CrG10H*, which implied it may have catalytic activity in geraniol hydroxylation process as in *C. roseus *(Additional file 4). SCS, the second CYP450, is the last enzyme in the biosynthesis of secologanin. Unigenes, assembled from 165 reads in our library, were annotated to the *CrSCS*. One putative *SCS *gene in *C. acuminata *(*CaPSCS*) was cloned (GenBank ID: HQ605982), according to a contig that had annotated to the *CrSCS *gene. The molecular weight of the predicted protein was approximately 60 kDa. Protein subcellular localization prediction using the WoLF PSORT program [34] indicated that the presumed protein was likely targeted to the endoplasmic reticulum (ER) membrane. Protein alignment revealed that the predicted protein shared 68% amino acid identity to that in *C. roseus*, which is involved in a similar terpenoid indole alkaloids biosynthetic pathway (Additional file 5). From the analysis, we inferred that the *CaPSCS *gene may play a role in secologanin biosynthesis in *C. acuminata*. STR is the enzyme that catalyzes the reaction of strictosidine synthesis. We cloned the ORF of a putative *STR *gene (*CaPSTR*, GenBank ID: JF508375) from *C. acuminata*. Phylogenetic analysis showed that STR proteins from reported alkaloid-producing plants were clustered together, which implied that the candidate gene possibly plays a role in CPT biosynthesis (Figure 3A).

#### Expression analysis of transcripts for proteins involved in strictosidine synthesis

Methyl jasmonate (MeJA)-induced accumulation of secondary metabolites and related gene expression has been reported in medicinal plants such as *Panax ginseng *and *C. roseus *[35-37]. A previous report determined that the CPT content responded to MeJA and jasmonic acid, and that the response curve for jasmonic acid treatment was a waveform, with two time-specific CPT accumulation peaks in *C. acuminata *suspension cells [38]. However, there are few reports of the effect of MeJA treatment on the expression of genes of CPT biosynthesis in *C. acuminata*. In response to MeJA treatment, transcripts of *G10H*, *SCS *and *STR *were regulated in a waveform manner, including two expression peaks during 24 hours of induction (Figure 3B). The trend of the curve was consistent with the result of a previous report for genes in anthocyanin biosynthesis [39]. In this study, all the detected genes responded to MeJA immediately, with a common peak within one hour of induction, and then decreased rapidly to even lower levels than the control. The expression levels increased again to the second peak, whose timing was gene-specific. Therefore, we speculate that transcripts of *CaG10H, CaSCS*, and *CaSTR *were most likely to be involved in CPT biosynthesis. It has been reported that TDC genes, which are responsible for the production of tryptamine for auxin and CPTs, do not respond to MeJA [40]. The expression of HMGR genes is even inhibited by MeJA in *C. acuminata *[41]. This is likely to be because the substrates of TDCs and HMGRs link primary and secondary metabolism, and their expression profiles are complicated.

Previous reports had shown that young and actively growing tissues, showed the highest level of CPT [26]. In this study, the mRNA levels of *CaG10H*, *CaSCS *and *CaSTR *were detected using real-time PCR. The results demonstrated that the expression levels of the three genes were all higher in young leaves and petioles than in old leaves (Figure 3C). Therefore, young leaves and young petioles are the possible sites of active CPT synthesis, as well as sites of accumulation, in *C. acuminata *compared with the mature tissues. This difference served as a standard for real-time PCR detection for downstream candidate gene selection [42]. Meanwhile, the expression levels of these genes were also relatively lower in the root and root bark which implied that root may not be a main synthetic tissue. This was consistent with the expression pattern of the *TDC1 *and *10HGO *genes reported in a previous study [16].

### Transcripts for proteins likely to be involved in the branch pathway of CPT synthesis

Strictosidine rapidly forms the intermediate product strictosamide in *C. acuminata*. The steps after strictosamide synthesis remain somewhat unclear. Based on the proposed branch steps, an intermediate step between strictosamide and 3(S)-pumiloside in the CPT biosynthetic pathway was presumed to be catalyzed by a cytochrome P450, with another P450 possibly in the last steps of CPT biosynthesis (Figure 1B). Cytochrome P450s, are a large and complex superfamily, which play important roles through catalysis of oxidation and hydroxylation reactions. In *C. acuminata*, no cytochrome P450 involved in the downstream CPT biosynthetic pathway had been cloned and identified. After EST annotation against the Swissprot database, 99 putative cytochrome P450 transcripts were identified in the 454 ESTs pool (Additional file 6), belonging to 28 cytochrome P450 subfamilies, according to the standard CYP family categories (Additional file 7). According to clan classification, transcripts of CYP71 clan and CYP72 subfamilies are likely to be involved in secondary metabolism [43]. A total of 27 cytochrome P450 transcripts belonging to these two subfamilies were discovered as candidate genes for further screening. Glucosidases, which is a superfamily involved in various biological process including cell wall assembly, polysaccharides, plant defense and secondary metabolism, catalyze the action of deglycosylation [44]. It had been reported that β-D-glucosidase plays a role in glycoside hydrolysis in TIA biosynthesis in plants such as *C. roseus *[11], *Psychotria ipecacuanha *[45] and *R. serpentine *[46,47]. In the 454 dataset, one transcript (contig 00133) annotated as strictosidine β-D-glucosidase in *C. roseus *(CrSGD) was identified with a predicted homologous peptide of 178 amino acids. The peptide was found to share 70% similarity to amino acids 47-224 of CrSGD and 62% similarity to amino acids 18-195 of the β-D-glucosidase IpeGlu1 of *Psychotria ipecacuanha *(PiIpeGlu1), which is involved in ipecac alkaloid synthesis. When compared with raucaffricine-O-beta-D-glucosidase (RsRD) and SGD (RsSGD) of another TIA-producing plant, *R. serpentine*, these proteins showed 69% and 74.8% (Additional file 8) similarity, respectively. CrSGD, RsRD, RsSGD and IpeGlu1 all belong to the glycosyl hydrolase (GH) family, which catalyzes the deglycosylation reaction in the TIA pathway, and their substrates are strictosidine, raucaffricine and N-deacetyli(so)pecoside, respectively. The predicted glucosidase peptide demonstrated high amino acid similarity with the glucosidases identified above as being involved in the alkaloid biosynthetic pathway. Therefore, CaPGD is likely to be a key enzyme in CPT synthesis through removal of a glucose moiety. Analysis of the amino acids of the predicted peptide demonstrated that three key amino acids 161-His, 207-Glu and 210-Thr, which were key amino acids for catalytic activity [47], are found in the corresponding residues of the predicted peptide of the *PGD *transcript in *C. acuminata*. The 388-Trp was not included in the peptide.

**Figure 4:**
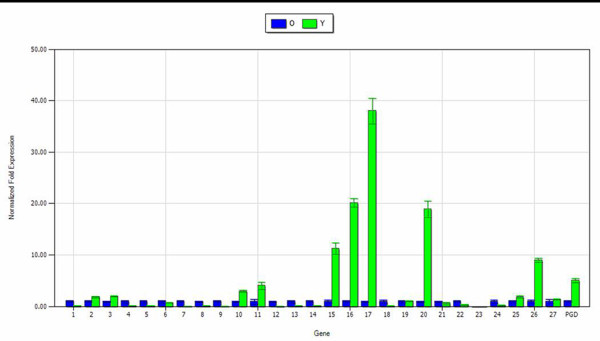
**Expression analysis of the cytochrome P450s and CaPGD transcripts in different tissues of the dataset**. The expression in old leaves was set to be the control. O: old leaves; Y: young leaves. 1-27 represent 27 cytochrome P450 transcripts in this dataset.

After initial screening, relative expression analysis in young/old leaves of the 27 cytochrome P450s and the CaPGD was performed for *C. acuminata*. Consequently, six cytochrome P450 transcripts and one CaPGD transcript that were shown to be expressed three-fold higher than the control were identified as candidate genes for participating in the branch pathway of CPT biosynthesis (Figure 4).

### Transcripts for proteins likely to participate in CPT transport

**Figure 5:**
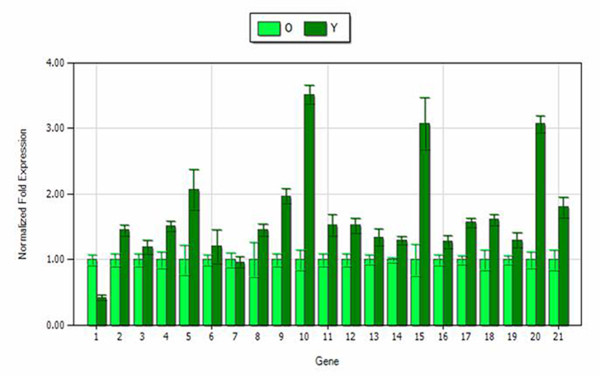
**Expression analysis of *MDR *transcripts in the dataset**. O: old leaves; Y: young leaves. The gene expression in old leaves was served as the control. 1-21 were 21 MDR transcripts in the annotated dataset.

MDR is a subfamily of the ABC transporter family that has been reported to be related to the transport of alkaloids metabolites [18]. From the annotated databases, 21 MDR transporters were found in the 132 ABC transporter transcripts in the library. Some of the transcripts were possibly responsible for CPT transport from synthesis site to the glandular trichomes in leaves through the plasma membrane [48]. Previous studies showed that the CPT content was four to five-fold higher in young *C. acuminata *leaves compared with mature leaves [49]. It is possible that CPT transporters were more abundant in the young leaves than in mature ones [50]. Subsequently, the 21 annotated MDR transporter transcripts were subjected to expression analysis in young leaves and old leaves of *C. acuminata *by real-time PCR. The results showed that the expression level of three transcripts (FXAT9O006HB5TT, FXAT9O006HKTK5, and contig05927) among the annotated MDR transporters were three-fold higher in young leaves than in the mature leaves. Thus, they represent candidate genes for CPT transportation in leaves (Figure 5).

### Probable site of CPT biosynthesis

Young leaves are the main site for CPT accumulation; therefore, this tissue was used to identify new genes in CPT biosynthetic pathway by high throughput sequencing. After assembly and annotation, 20 enzyme genes that act before the step of strictosidine synthesis were found in the dataset, including the key genes encoding G10H, SCS and STR. This result indicated that many putative genes in CPT synthesis are expressed in young leaves, which demonstrates that young leaves are likely to be active tissues for CPT biosynthesis as well as accumulation. Expression profile analysis indicated that the biosynthesis of strictosidine may be more active in young leaves and petioles than in mature leaves and roots. This result indicates that CPT is likely to be synthesized in young leaves, which is consistent with a recent study [16]. Young leaves and petioles are likely to be the main sites for CPT biosynthesis. The lower expression in roots implies that roots may not be a main tissue for CPT biosynthesis; however, CPT does accumulate in roots. Therefore, our results support the hypothesis that in *C. acuminata*, the main CPT synthesis site is the young leaf.

The subcellular site for CPT synthesis in *C. acuminata*, has not been reported previously. In this study, we predicted that the *CaSCS *gene was localized in the ER. The results indicated that secologanin in *C. acuminata *is possibly biosynthesized in the ER, which was consistent with the studies of CPT location in hairy roots of *O. pumila *[48]. Therefore, we hypothesize that in *C. acuminata *CPT is likely to be biosynthesized in the ER and then transported to a vacuole [15] or excreted outside the cytoplasmic membrane, as reported in *O. pumila *[51].

## Conclusion

In this study, a high quality cDNA library was established to mine effective transcriptome information in CPT biosynthesis and transport in *C. acuminata*. A method was adopted for gene discovery using a combination of sequence annotation, chemical catalytic features prediction and transcripts expression profiling for deep mining of target genes of the CPT metabolism pathway. Consequently, a number of putative transcripts, including genes encoding G10H, SCS, STR, cytochrome P450s, glucosidase, and MDR transporter genes, were identified as possibly being involved in CPT biosynthesis and transport. Meanwhile, three important genes encoding proteins involved in CPT backbone biosynthesis were cloned and analyzed. The transcriptome data represents a valuable genetic resource for further identification of genes involved in CPT biosynthesis and transport. This dataset could be beneficial for further research of the CPT metabolism pathway and molecular genetic breeding.

## Methods

### Materials preparation and treatment

Young leaves (the first leaf from the apex of side branches, including the apex) for library construction and gene cloning [52] were collected from a *C. acuminata *tree with a diameter of 14 cm cultivated in the greenhouse of the IMPLAD (Institute of Medicinal Plant Development), Beijing, China. Root, root bark, stem, petiole (the petiole of the first young leaf), young leaves (the first leaf from the apex of the side branches) and old leaves (the fifth leaf from the apex of the side branches) were prepared from the same tree for expression analysis as described previously [52]. The second young leaves from the apex of each branch (about 4 cm long) were cut off for treatment. For the MeJA induction experiment, young leaves were soaked in 100 μM MeJA, with unsoaked leaves serving as a control. The experimental materials were then immediately frozen in liquid nitrogen and stored at -80°C for further processing. All the real-time experiments were repeated three times.

### RNA preparation

Total RNA was isolated using the Universal Plant RNA Isolation Mini Kit (BioTeke, Beijing, China), according to the manufacturer's recommendation. Total RNA quantity and quality were determined with a GeneQuant100 spectrophotometer (GE Healthcare, UK) and 1% agarose gels.

### cDNA library construction

Total RNA was extracted from young leaves of *C. acuminata*. RNA samples were digested with RNase-free DNase I (TURBO DNase; Ambion, TX, USA) immediately after RNA extraction. The digested RNA was converted to cDNA using a SMART cDNA synthesis kit (Clontech, CA, USA) and then amplified by applying the Advantage II polymerase (Clontech, USA) to increase the total quantity of the sample for sequencing. Purification of the amplified products was carried out with the PureLink™ PCR purification kit (Invitrogen, USA). Sequences shorter than 300 bp were removed, and approximately 5 μg purified cDNA was sent for a 1/8 run using the 454 GS FLX platform shotgun sequencing (454 Life Sciences, Roche).

### EST assembly

GS FLX *De Novo *Assembly Software v2.0.01 (454 Life Sciences, Roche) was used for EST processing and assembly. ESTs with weak signals and low quality were filtered through the software analysis (using default parameters). Sequencing adaptors were trimmed using the software, and then high-quality (> 99.5% accuracy on single base reads) reads were generated (using default parameters). The SMART PCR primers (Clontech) were then screened, and HQ reads that were shorter than 50 bp were removed for data cleaning of the cDNA library. The remaining HQ ESTs were used for *de novo *assembly using the GS FLX *De Novo *Assembly Software v2.0.01 (using default parameters), with a quality score threshold set at 40. After assembly, all the sequences, including contigs (obtained from one cluster) and singletons (appeared only once), were named as "unigenes" for subsequent annotation.

### Functional annotation and classification

Similarity searches were carried out against a series of nucleotide and protein databases, such as the Nt, Nr, SwissProt, Kegg, and TAIR databases [28-32], with a common significance threshold cutoff of *E*-value ≤ 1e-5. For the database annotation, the top five results based on BLAST scores were retained for transcriptome analysis. Gene Ontology classification of TAIR was used to assign the functional roles of *C. acuminata *through similarity searches. All unigenes were classified into forty-five subcategories belonging to three major categories: cellular component, molecular function and biological process.

In this study, the transcripts were identified and screened by searching the annotation for scores over 100 and were checked manually.

### ORF cloning of putative genes encoding proteins from the backbone of CPT biosynthesis

RNA samples of young leaves for gene cloning were converted to first-strand cDNA of the 5' and 3' ends according to the SMART™ RACE cDNA Amplification Kit User Manual (Clontech, USA). RACE PCR Primers for *G10H *and *STR *cloning were designed based on the sequence of FXAT9O006GXSI6 and contig03632 respectively in the dataset (Table S1). Primers for *G10H *and *STR *genes cloning were designed according to the entire assembled sequence of RACE PCR. Gene cloning of *SCS *was performed using the annotated unigene contig00661 in the cDNA library, which had integrated ORF sequences. Primers for *SCS *cloning were designed from the 3' end and 5' untranslated region of contig 00661, which contained an entire ORF. Advantage 2 Polymerase Mix (Clontech, USA) was used for PCR amplification of 3' ends, 5' ends and ORFs of the three genes. All three genes were amplified at 95°C for 3 min; followed by 25 cycles of 95°C for 30 sec, 57°C for 30 sec and 72°C for 1 min 30 sec; and a final step at 72°C for 10 min. The recycled products were integrated into a pMD^® ^18-T vector (Takara, Dalian, China) and transferred into *E. coli *DH5α competent cells (Transgene, Beijing, China). The isolated clones were sequenced on a 3730XL (ABI, USA). Sequence alignment with *CrG10H and CrSCS *in *C. roseus *was carried out using the DNAMAN software (Lynnon Biosoft, USA). A phylogenetic tree of CaPSTR was constructed according to the amino acid sequences of selected plants. The evolutionary analysis was generated using the software of MEGA 4.0.

### Expression analysis

To determine the expression profile of the transcripts involved in CPT biosynthesis, mRNA levels of the transcripts at different tissues and under different treatments were analyzed using Quantitative Real-time PCR. The PrimeScript™ 1st Strand cDNA Synthesis Kit (TaKaRa, Dalian, China) was used for single-strand cDNA synthesis using 1 μg RNase-free DNase I-treated (TaKaRa, Dalian, China) total RNA. Quantitative PCR (Q-PCR) was carried out at least three times each with SYBR^® ^Premix Ex TaqTM (Perfect Real Time) (TaKaRa, Dalian, China) on an IQ5 Multicolor Real-Time PCR Detection System (Bio-Rad, USA). Each qRT-PCR system contained 10 μL 2 × SYBR^® ^Premix Ex Taq™, 0.2 μM forward and reverse primers and 1 μL cDNA template. The PCR amplification program was as follows: 50°C for 2 min; 95°C for 30 sec; 40 cycles of 95°C for 3 sec and 62°C for 40 sec; followed by a melting-curve program of 55°C to 85°C, with a 5-sec hold at each temperature. The gene expression patterns of all genes were normalized to an internal reference (*18S *rRNA) [53]. The relative gene expression analysis was performed using BIO-RAD IQ™5 optical system software version 2.0 with the 2^-ΔΔCt ^method. All the real-time PCR primers were designed using OMIGA software (Accelrys, USA) with suitable parameters (length: 100-300 bp; Tm: approximately 62°C). The sequences of all primers are listed in the supporting information (Additional file 9).

## Authors' contributions

YZS contributed to collect experiment samples, designed and carried on the experiment of post sequencing and drafted the manuscript. HML prepared the 454-library and helped to modify the draft. YL, YJZ analyzed the data. YYN, CS, JYS and TE helped to modify the manuscript. LD helped to make the chemical draw. APL and SLC initiated the project, helped to conceive the study and participated in the design and coordination. All authors had read and approved the final manuscript.

## Acknowledgements

This study was supported by the Program for the National Key Technology R&D Foundation of China (No.2006BAI09B05-3, 81130069) and the National Natural Science Foundation of China (30970307 and 30900113). We also thank Professor Yu-Lin Lin (Institute of Medicinal Plant Development, Chinese Academy of Medical Sciences, China) for his kind help in the identification of the plant of *C. acuminata*.

## Supplementary Material

Additional file 1 **Annotation statistics against public databases**. Word document for the summary of the annotation result. Click here for file

Additional file 2 **Gene Ontology analysis of the 454 sequencing library**. TIFF document for the function categorization of the library against the Arabidopsis database. Click here for file

Additional file 3 **Gene discoveries for CPT biosynthesis against the Nr, Swissprot and Kegg databases**. Excel document of specific information for mining genes in CPT biosynthesis. Click here for file

Additional file 4 **Amino acid alignment between CaG10H and CrG10H**. TIFF document of protein sequence alignment of CaG10H and CrG10H. Click here for file

Additional file 5 **Peptide alignment between CaSCS and CrSCS**. TIFF document of protein sequence alignment of CaSCS and CrSCS. Click here for file

Additional file 6 **Transcripts of CYP450s discovered in this dataset**. Excel document of all the discovered transcripts of cytochrome P450. Click here for file

Additional file 7 **Classification of transcripts annotated to cytochrome P450s in this library**. Word document of the classification of cytochrome P450s transcripts. Click here for file

Additional file 8 **Amino acid alignment between the predicted CaPGD and RsSGD**. TIFF document of the comparison of CaPGD and RsSGD. Click here for file

Additional file 9 **Primers used in this study**. Excel document of all the designed primers in this study. Click here for file
