# Supplementary material for: Pyrosequencing of the Camptotheca acuminata transcriptome reveals putative genes involved in camptothecin biosynthesis and transport
Source: BMC Genomics. 2011 Oct 30;12:533. doi: 10.1186/1471-2164-12-533 (PMC3229617; doi:10.1186/1471-2164-12-533)
Supplement: Additional file 1 — Annotation statistics against public databases. Word document for the summary of the annotation result. [file 1471-2164-12-533-S1.DOC]

**Table S1A Summary of annotation statistics against public databases.**

| **Library** | **Unigenes** | **Annotaed** | **Percent(%)** | **Unannotated** | **Percent(%)** |
| --- | --- | --- | --- | --- | --- |
| Nr | 30358 | 20079 | 66.1 | 10279 | 33.9 |
| Kegg | 30358 | 15753 | 51.9 | 14605 | 48.1 |
| Nt | 30358 | 18539 | 61.1 | 11819 | 38.9 |
| Swissprot | 30358 | 9733 | 32.1 | 20625 | 67.9 |
| TAIR | 30358 | 18172 | 59.8 | 12186 | 40.2 |

**Table S1B The top twenty abundant transcripts in the young leaf library in *Camptotheca acuminata*.**

| **contigs** | **Reads number** | **Annotation** |
| --- | --- | --- |
| contig09070 | 426 | Chlorophyll a-b binding protein 21 (Swissprot） |
| contig08875 | 392 | Chlorophyll a-b binding protein 21 (Swissprot） |
| contig09119 | 374 | Chlorophyll a-b binding protein 21 (Swissprot） |
| contig08913 | 234 | hypothetical protein (Kegg) |
| contig09025 | 216 | Ribulose bisphosphate carboxylase small chain (Swissprot） |
| contig09015 | 209 | Agglutinin (Swissprot） |
| contig00386 | 197 | Ubiquitin (Swissprot） |
| contig09120 | 193 | No annotation |
| contig01281 | 188 | Peroxidase 42 (Swissprot） |
| contig08985 | 182 | No annotation |
| contig09113 | 176 | Chlorophyll a-b binding protein 21 (Swissprot） |
| contig00033 | 176 | UPF0580 protein C15orf58 homolog (Swissprot） |
| contig01920 | 169 | Chlorophyll a-b binding protein 7 (Swissprot） |
| contig01275 | 169 | Oxygen-evolving enhancer protein 1 (Swissprot） |
| contig00119 | 162 | Chlorophyll a-b binding protein CP26 (Swissprot） |
| contig09044 | 159 | Glyceraldehyde-3-phosphate dehydrogenase B (Swissprot） |
| contig00115 | 153 | Chlorophyll a-b binding protein 151 (Swissprot） |
| contig09088 | 147 | No annotation |
| contig00743 | 138 | 21 kDa seed protein (Swissprot） |
| contig00382 | 138 | EG45-like domain containing protein (Swissprot） |

**Table S1C The top ten longest transcripts discovered in the 454 library in *Camptotheca acuminata*.**

| **Contigs** | **Length(bp)** | **Annotation** |
| --- | --- | --- |
| contig01236 | 3848 | magnesium-chelatase H subunit (Swissprot） |
| contig00530 | 3669 | Glycine dehydrogenase (Swissprot） |
| contig02014 | 3206 | Ferredoxin-dependent glutamate synthase (Swissprot） |
| contig01943 | 3093 | Eukaryotic translation initiation factor 3 subunit A (Swissprot） |
| contig02355 | 2942 | Probable alpha, alpha-trehalose-phosphate synthase (Swissprot） |
| contig00004 | 2932 | predicted protein [Populus trichocarpa] (Nr) |
| contig02619 | 2925 | Uncharacterized glycosyltransferase (Swissprot） |
| contig00331 | 2841 | Probable UDP-N-acetylglucosamine--peptide N-acetylglucosaminyltransferase (Swissprot） |
| contig00628 | 2835 | Aminopeptidase (Swissprot） |
| contig01032 | 2799 | Putative ion channel CASTOR, chloroplastic (Swissprot） |
