# Supplementary figures and images for: Pyrosequencing of the Camptotheca acuminata transcriptome reveals putative genes involved in camptothecin biosynthesis and transport
Source: BMC Genomics. 2011 Oct 30;12:533. doi: 10.1186/1471-2164-12-533 (PMC3229617; doi:10.1186/1471-2164-12-533)

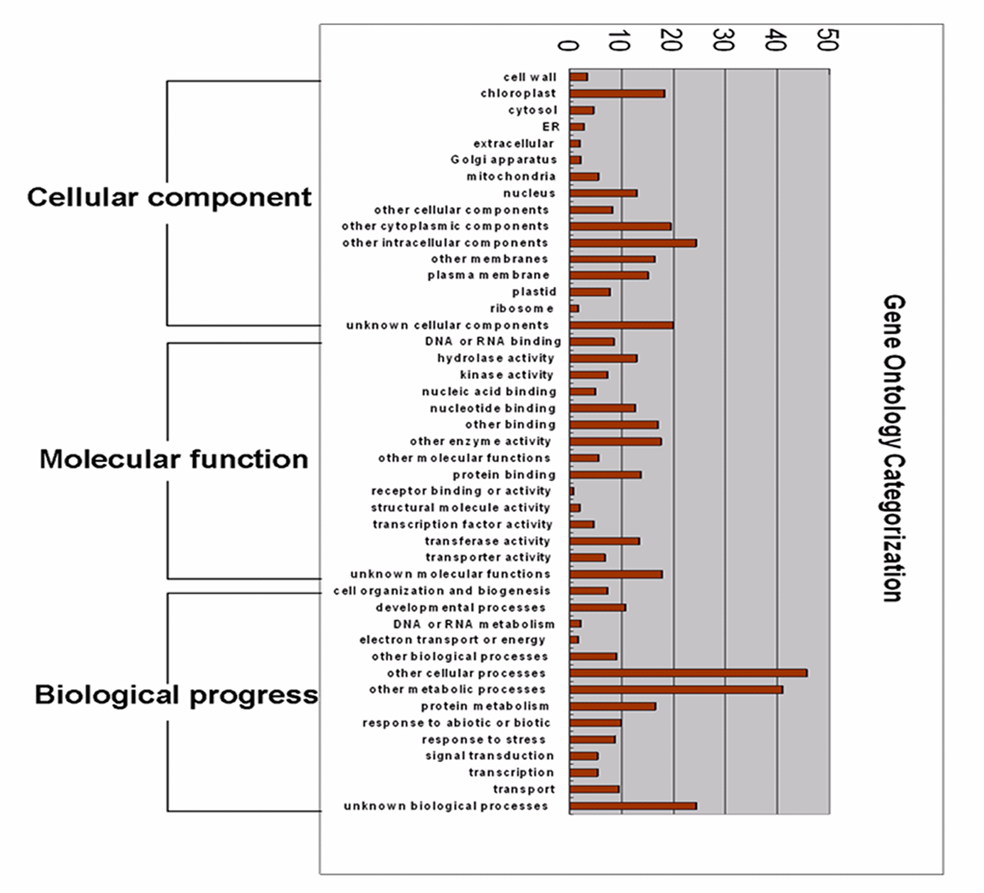

Supplement: Additional file 2 — Gene Ontology analysis of the 454 sequencing library. TIFF document for the function categorization of the library against the Arabidopsis database. [file 1471-2164-12-533-S2.TIFF]

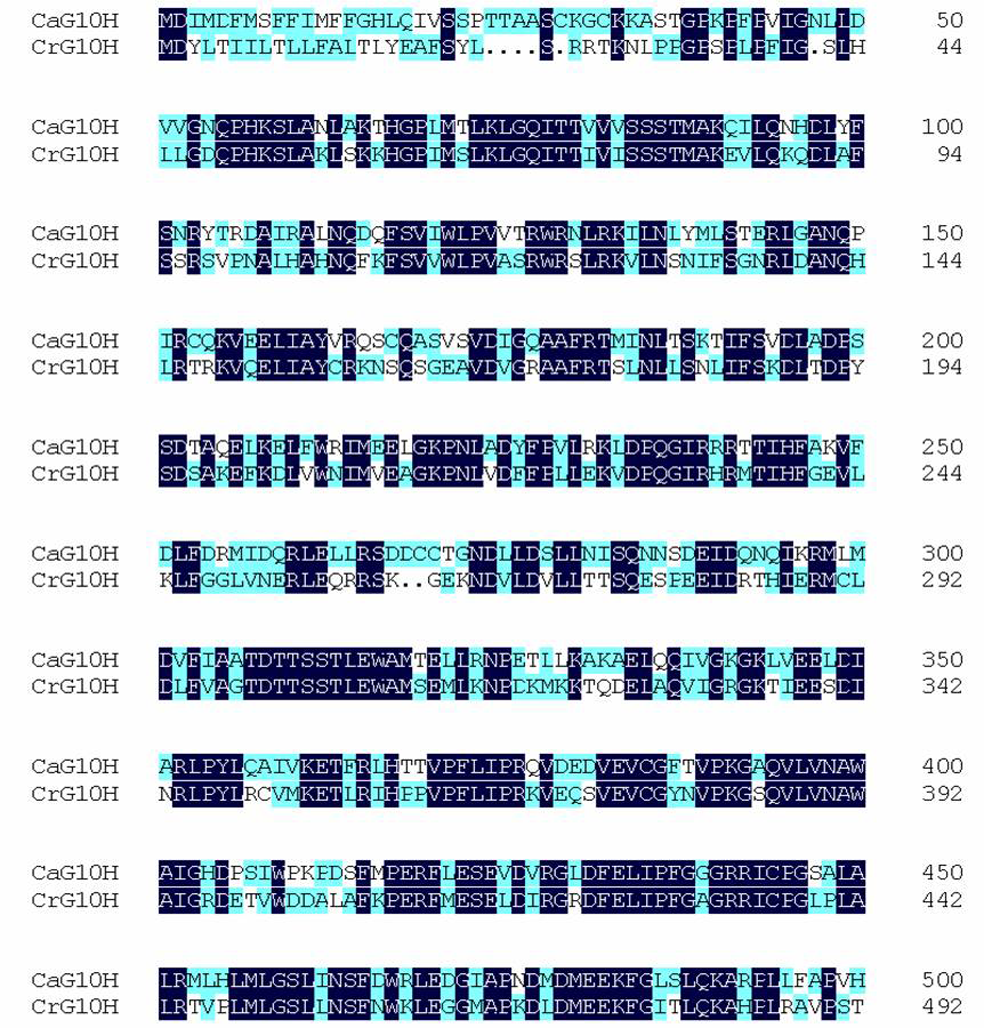

Supplement: Additional file 4 — Amino acid alignment between CaG10H and CrG10H. TIFF document of protein sequence alignment of CaG10H and CrG10H. [file 1471-2164-12-533-S4.TIFF]

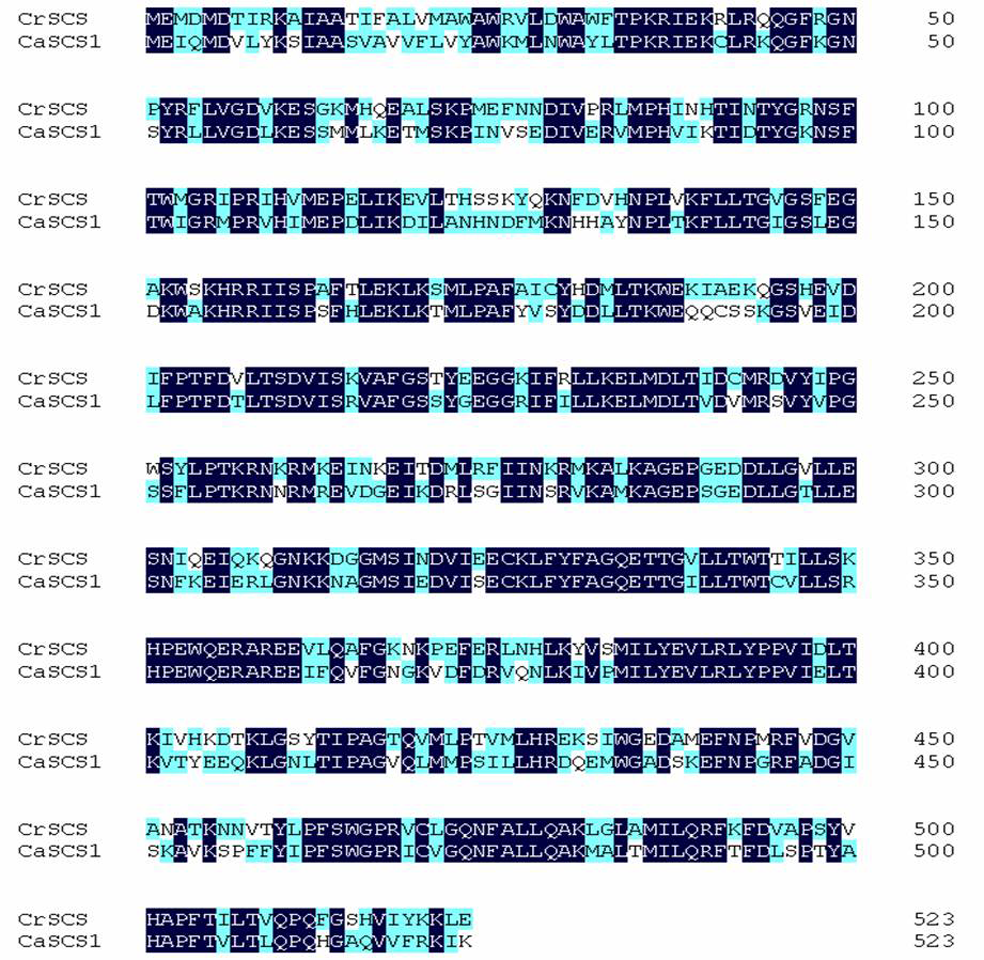

Supplement: Additional file 5 — Peptide alignment between CaSCS and CrSCS. TIFF document of protein sequence alignment of CaSCS and CrSCS. [file 1471-2164-12-533-S5.TIFF]

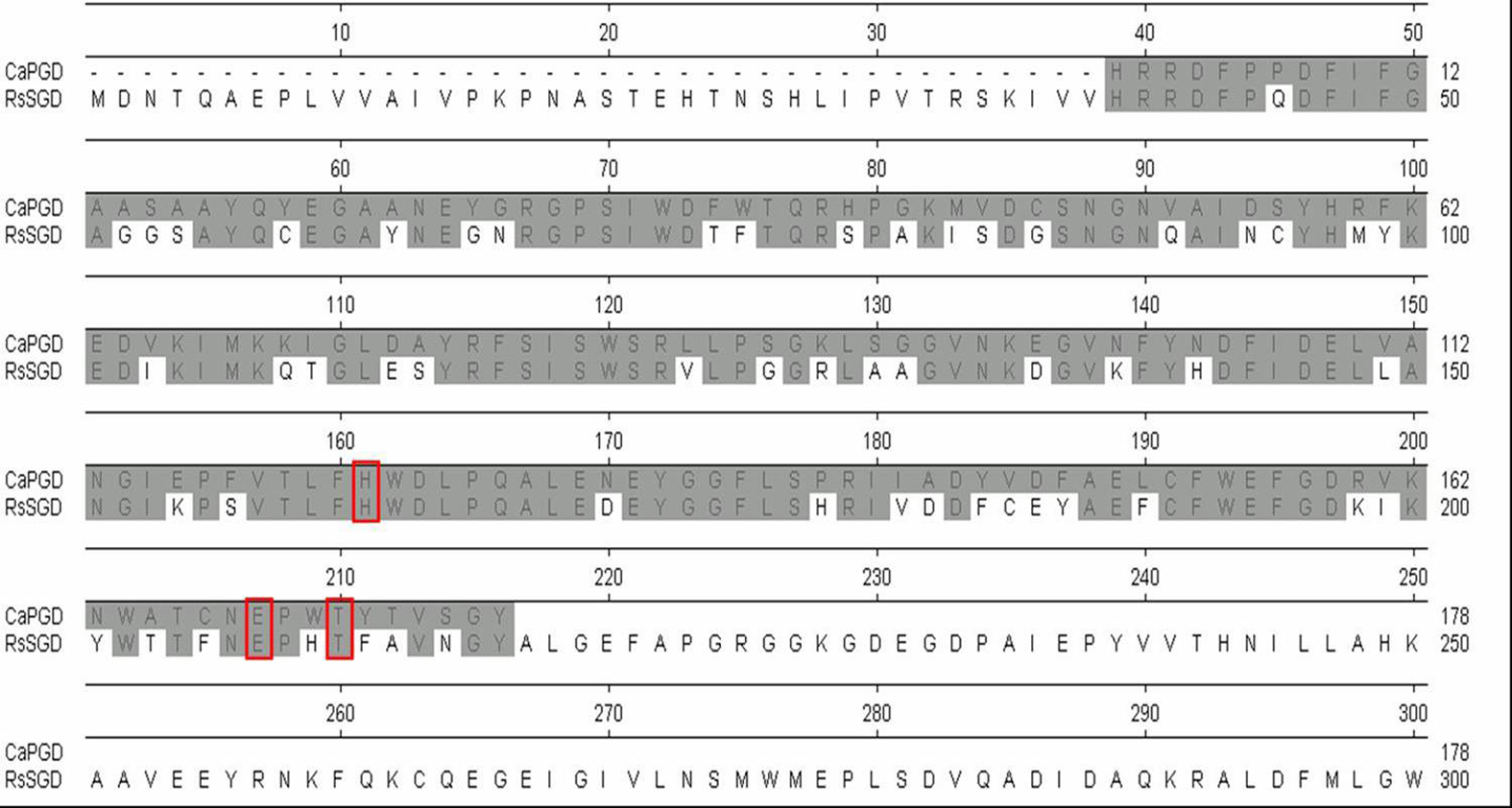

Supplement: Additional file 8 — Amino acid alignment between the predicted CaPGD and RsSGD. TIFF document of the comparison of CaPGD and RsSGD. [file 1471-2164-12-533-S8.TIFF]
