# Supplementary material for: Pyrosequencing of the Camptotheca acuminata transcriptome reveals putative genes involved in camptothecin biosynthesis and transport
Source: BMC Genomics. 2011 Oct 30;12:533. doi: 10.1186/1471-2164-12-533 (PMC3229617; doi:10.1186/1471-2164-12-533)
Supplement: Additional file 7 — Classification of transcripts annotated to cytochrome P450s in this library. Word document of the classification of cytochrome P450s transcripts. [file 1471-2164-12-533-S7.DOC]

### Table S2 Classification of the candidate P450 genes against Swissprot database

| **CYP subfamilies** | **No. of subfamily** | **Unigenes number** | **Total EST number** | **No. of 454EST** |
| --- | --- | --- | --- | --- |
| CYP3 | 1 | 1 | 4 | contig01746 |
| CYP4 | 2 | 2 | 4 | FXAT9O006G80PS, contig01103 |
| CYP51 | 1 | 3 | 5 | FXAT9O006G4MG9, FXAT9O006HC9T3,  contig03572 |
| CYP71 | 6 | 7 | 10 | FXAT9O006G0J13,FXAT9O006GTQEA,FXAT9O006G7MF2, FXAT9O006G3PTZ, XAT9O006GTQTW FXAT9O006G2WHB, contig07510 |
| CYP72 | 1 | 17 | 165 | FXAT9O006G6ISW,FXAT9O006G8IDN,FXAT9O006G8RH9,FXAT9O006GW8Y6,FXAT9O006GZ0LG,FXAT9O006GZ4SY,FXAT9O006GZDED,FXAT9O006HIS44,FXAT9O006HIVXN,FXAT9O006HK0MG,contig00661,contig01536,contig01540,contig01903,contig02128,contig02902,contig07956 |
| CYP73 | 3 | 3 | 11 | FXAT9O006GSWLH,contig01080,contig00168 |
| CYP74 | 2 | 5 | 25 | FXAT9O006HK2T2, FXAT9O006GXTUF,  FXAT9O006HHRUJ,contig01073, contig01863 |
| CYP75 | 1 | 1 | 1 | FXAT9O006GRIYW |
| CYP76 | 3 | 5 | 12 | FXAT9O006GXSI6，FXAT9O006G9LTF, FXAT9O006GXSI6, contig06009, FXAT9O006G0OXA, contig07696 |
| CYP77 | 2 | 2 | 5 | contig05921, contig01429 |
| CYP78 | 1 | 3 | 4 | FXAT9O006G3ZG1,FXAT9O006GQ52W,  contig04589 |
| CYP80 | 1 | 1 | 1 | FXAT9O006G8NK9 |
| CYP81 | 3 | 7 | 7 | FXAT9O006G3FW7,FXAT9O006HKFL7,  FXAT9O006HLX5O,FXAT9O006HCGEW,  FXAT9O006G3KFO,FXAT9O006HH8NS,  FXAT9O006HHSY9 |
| CYP82 | 1 | 2 | 3 | FXAT9O006GWCU5, contig07889 |
| CYP84 | 1 | 2 | 2 | FXAT9O006HBDBI, FXAT9O006HBLOG |
| CYP85 | 2 | 2 | 6 | contig08494, contig05837 |
| CYP86 | 2 | 4 | 5 | FXAT9O006G3G2F, FXAT9O006HMAZ1,  FXAT9O006GW27E, contig05799 |
| CYP89 | 1 | 1 | 1 | FXAT9O006HH5E3 |
| CYP90 | 1 | 3 | 6 | FXAT9O006G0GFD，FXAT9O006HHG74，contig04845，FXAT9O006G80A4, FXAT9O006HDN9T, contig07076 |
| CYP93 | 2 | 3 | 16 | contig08006,contig02048,contig03635 |
| CYP94 | 2 | 3 | 3 | FXAT9O006G6LTD, FXAT9O006HFN6L,  FXAT9O006HMP06 |
| CYP97 | 1 | 4 | 27 | FXAT9O006G57KJ,contig00416,contig02314,  contig07870 |
| CYP98 | 1 | 3 | 10 | FXAT9O006G0QN0, FXAT9O006HDYQ6,  contig03356 |
| CYP704 | 1 | 2 | 4 | FXAT9O006G9OEV, FXAT9O006HB0NX |
| CYP707 | 2 | 2 | 7 | contig08551, contig05836 |
| CYP716 | 2 | 5 | 8 | contig05161, FXAT9O006G0ASF,  FXAT9O006G1MAT,FXAT9O006GWM6B,  FXAT9O006GYH0D |
| CYP724 | 1 | 1 | 1 | FXAT9O006G8VX7 |
| CYP750 | 1 | 1 | 1 | FXAT9O006GWUFE |
| total | 48 | 95 | 358 | 95 |
